# *TARE1*, a Mutated *Copia*-Like LTR Retrotransposon Followed by Recent Massive Amplification in Tomato

**DOI:** 10.1371/journal.pone.0068587

**Published:** 2013-07-04

**Authors:** Hao Yin, Jing Liu, Yingxiu Xu, Xing Liu, Shaoling Zhang, Jianxin Ma, Jianchang Du

**Affiliations:** 1 Bioinformatics Group, Institute of Industrial Crops, Jiangsu Academy of Agricultural Sciences, Nanjing, China; 2 Center of Pear Engineering Technology Research, State Key Laboratory of Crop Genetics and Germplasm Enhancement, Nanjing Agricultural University, Nanjing, China; 3 Department of Agronomy, Purdue University, West Lafayette, Indiana, United States of America; Institut Pasteur, France

## Abstract

Long terminal repeat retrotransposons (LTR-RTs) are the major DNA components in flowering plants. Most LTR-RTs contain dinucleotides ‘TG’ and ‘CA’ at the ends of the two LTRs. Here we report the structure, evolution, and propensity of a tomato atypical retrotransposon element (*TARE1*) with both LTRs starting as ‘TA’. This family is also characterized by high copy numbers (354 copies), short LTR size (194 bp), extremely low ratio of solo LTRs to intact elements (0.05∶1), recent insertion (most within 0.75∼1.75 million years, Mys), and enrichment in pericentromeric region. The majority (83%) of the *TARE1* elements are shared between *S. lycopersicum* and its wild relative *S. pimpinellifolium*, but none of them are found in potato. In the present study, we used shared LTR-RTs as molecular markers and estimated the divergence time between *S. lycopersicum* and *S. pimpinellifolium* to be <0.5 Mys. Phylogenetic analysis showed that the *TARE1* elements, together with two closely related families, *TARE2* and *TGRE1*, have formed a sub-lineage belonging to a *Copia*-like *Ale* lineage. Although *TARE1* and *TARE2* shared similar structural characteristics, the timing, scale, and activity of their amplification were found to be substantially different. We further propose a model wherein a single mutation from ‘G’ to ‘A’ in 3′ LTR followed by amplification is responsible for the origin of *TARE1*, thus providing evidence that the proliferation of a spontaneous mutation can be mediated by the amplification of LTR-RTs at the level of RNA.

## Introduction

Retrotransposons are a class of transposable elements (TEs), which initiate their transposition through a copy-and-paste mechanism via RNA intermediates [1]. Retrotransposons can be divided into at least five orders on the basis of their structural features, namely, long terminal repeat retrotransposons (LTR-RTs), *Dictyostelium intermediate repeat sequence* (*DIRS*)-like elements, *Penelope*-like elements (PLEs), LINEs and SINEs [2]. Among these, LTR-RTs are the major genomic components of plants, particularly in species with complex genomes. For example, approximately, 20% of rice genome [3], 42% of soybean [4], 55% of sorghum [5], and over 75% of the maize genomes [6] are composed of LTR-RTs.

A typical intact LTR-RT element contains two identical LTRs, a primer-binding site (PBS), a polypurine tract (PPT), as well as *gag* and *pol*, two genes necessary for transpositional process [1]. LTRs terminate in short inverted repeats, usually 5′-TG-3′ and 5′-CA-3′, and they can be further divided into three parts, including U3, R and U5 [1]. Since two LTRs of an element are identical at the time of insertion, the insertion time of an element can be roughly converted by the sequence divergence of two LTRs if an appropriate mutation rate is employed [7]. For instance, the majority of LTR-RTs in soybean were amplified within the last one million years (Mys) [8]. The majority of LTR-RTs can be classified into *Copia*-like and *Gypsy*-like superfamilies based on the order of integrase (*int*), reverse transcriptase (*rt*) and RNase H (*rh*) in *pol* [9]. While some LTR-RT families are randomly dispersed in the host genome, most are concentrated in the recombination-suppressed pericentromeric regions [10]. Moreover, a few *Gypsy*-like LTR-RT families were found to be specific or enriched in centromeric regions, such as *CRR* elements (*CRR1* and *CRR2*) in rice [11], [12], *CRM* elements (*CRM1*, *CRM2*, and *CRM3*) in maize [12], and two families (*Gmr12* and *Gmr17*) in soybean [8]. Centromeric retrotransposons are considered to play an important role in plant centromere evolution and function [13].

In addition to intact elements, solo LTRs and truncated elements are another two forms of structural variations of LTR-RTs, and are usually dispersed in plant genomes [8], [14], [15]. These incomplete elements, together with numerous LTR remnants are presumed to be the products of unequal recombination and illegitimate recombination, which are two molecular mechanisms counterbalancing genome expansion [14], [15]. For instance, it was estimated that >190 Mb of DNA had been removed from the rice genome, leaving the current rice genome ∼400 Mb with ∼97 Mb DNA of detectable LTR-RTs [15].

Tomato (*Solanum lycopersicum*) is a major vegetable plant and is an ideal model system for studying fruit development [16]. The availability of high-quality genome sequence of cultivated tomato *S. lycopersicum* and the release of the draft genome of its wild relative *Solanum pimpinellifolium*, provides unprecedented opportunities for comparative analysis of transposable elements, evolutionary history, and domestication process in this important *Solanum* species. Using several sequenced BAC clones, two tomato LTR-RT families have been identified and characterized in previous studies, including *Gypsy*-like *Jinling* [17], and *Copia*-like *Rider* elements [18], [19], [20]. In the present study, we have identified and annotated >12,000 LTR-RT elements by screening the assembled genome sequence of cultivated tomato *S. lycopersicum*. Among these, one family, designated as *TARE1*, was of special interest because (1) the intact elements in this family have both LTRs starting as ‘TA’ instead of typical ‘TG’; (2) this family contains very short LTRs (194 bp), and the ratio of solo LTRs to intact element is extremely low (0.05∶1), supporting the idea that larger LTRs may facilitate solo LTRs formation; (3) over 60% elements were inserted into the genome ∼0.75–1.75 million years ago (Mya), rather than <1 Mys observed for most families in other species; (4) the elements in this family were amplified in *S. lycopersicum*, most of which can be found in *S. pimpinellifolium*, but not in other *Solanum* species; (5) we used shared elements as DNA markers to estimate the divergence time (<0.5 Mya) between *S. lycopersicum* and *S. pimpinellifolium* from their common ancestor; (6) a single mutation from ‘G’ to ‘A’ in 3′ LTR followed by amplification, were found to be responsible for the formation of the atypical structure in this family. Therefore, this study is the first comprehensive investigation of a single tomato LTR-RT family at a whole genome-wide level, and the data obtained provide insights into the evolution, divergence and domestication process between *S. lycopersicum* and its wild relative *S. pimpinellifolium*.

## Results

### Identification and Sequence Analysis of the *Copia*-like Retrotransposon *TARE1* in the Tomato Genome

**Figure 1:**
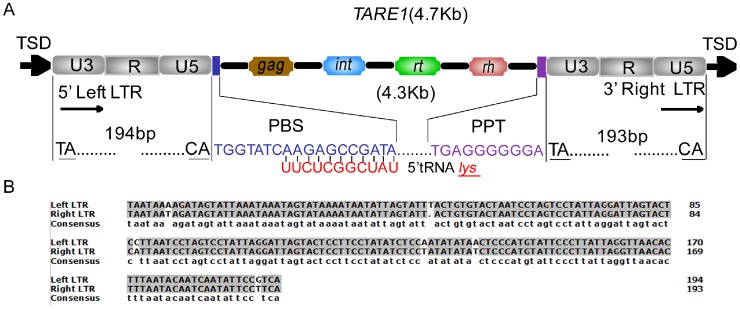
Schematic *TARE1* and the LTR sequence comparison. (A) Structural annotation for the *TARE1* element. The U3, R and U5 regions of LTR (Long terminal repeat) are shown in gray boxes; ‘TSD’ indicates the 5-bp target site duplication; ‘PBS’ means the primer binding site; ‘PPT’ indicates the polypurine tract; *int*, *rt* and *rh* are the abbreviations for integrase, reverse transcriptase and RNAase-H, respectively. (B) The sequence alignment of two LTRs from a randomly selected intact *TARE1* element. The identical nucleotides are shown with gray shadow. The insertions/deletions are marked by dots. The physical positions of this element are located at Chromosome 1 from 27235875 to 27240535.

Initially, 18 *TARE1* LTR-RTs were identified by the program LTR_STRUC [21]. However, the boundaries of these elements were found to be incorrect and misannotated. The *TARE1* sequence with its two flanking sequences (1 kb for each site) when combined with another and aligned, showed an accurate insertion site flanked by a perfect 5-bp target site duplication (TSD), an important signature for LTR-RT insertion. A typical *TARE1* LTR-RT has an element size ∼4.7 kb with two short LTR sequences (194 bp), a primer binding site (PBS) with the sequence ‘TGGTATCAAGAA’, a polypurine tract (PPT) site with a conserved motif ‘TGAGGGGGGA’, as well as *gag* and *pol* genes in the internal region (Figure 1A and Figure S1). The order of *int*, *rt* and *rh* within the *pol* defined *TARE1* as a *Copia*-like element (Figure 1A). We also found that most two LTRs for each *TARE1* element had accumulated a few mutations (Figure 1B), indicating that these elements inserted into the tomato genome previously. It is noteworthy that both LTRs of the *TARE1* element terminate by the two dinucleotides 5′-TA.CA-3′, instead of 5′-TG.CA-3′ usually found in previous studies [1].

### Structural Characterization of *TARE1* LTR Retrotransposons in the Tomato Genome

We were curious to elucidate the structure of *TARE1* elements at a genome-wide level. By using a combination of structure-based and homology-based approaches, as previously described [4], [15], we mined 760 Mb of assembled tomato genomic sequence for *TARE1* elements [16]. We found that this family contained 354 copies, including 180 intact elements with target site duplication (TSDs), 12 intact elements without TSDs, 10 solo LTRs with TSDs, 7 solo LTR without TSDs, and 145 truncated elements with at least one LTR that was partially deleted (Table 1 and Table S1). These elements, together with numerous related unrecognizable fragments, make up 5.6 Mb of DNA, accounting for ∼1% of the assembled tomato genomic sequence.

**Table 1:** Structure of LTR Retrotransposons identified in tomato.

Structure	No. of elements
Intact elements with TSDs	180
Intact elements without TSDs	12
Solo LTRs with TSDs	10
Solo LTRs without TSDs	7
Truncated elements with 5' end deleted	67
Truncated elements with 3' end deleted	45
Truncated elements with both 5' and 3' ends deleted	33
Total	354

Of the 354 *TARE1* elements, only 17 (∼5%) are solo LTRs. The ratio of solo LTRs to intact elements (with TSDs) is ∼0.05∶1 (Table 1), which is much lower than in *Arabidopsis* (1.16∶1) [14], rice (1.46∶1) [15], and soybean (1.29∶1) [8]. This low ratio perhaps represents the lowest value for a single LTR-RT family reported so far. This ratio is also much lower than that for *Jingling* elements (0.71∶1) and *Rider* elements (0.92∶1) in tomato [19], indicating that the low ratio of solo LTRs to intact elements is family-dependant rather than species-specific in tomato. Since the formation of solo LTRs was presumed to be the products of unequal homologous recombination between two LTRs of a single element [14], [15], the short LTRs (194 bp, Figure 1) of intact *TARE1* elements may inhibit the solo LTRs formation. This result corroborated our previous report in soybean that the ratio of solo LTRs to intact elements is positively correlated with LTR sizes [8].

### *TARE1* Elements are Enriched in Pericentromeric Heterochromatin but not in Centromeres

**Figure 2:**
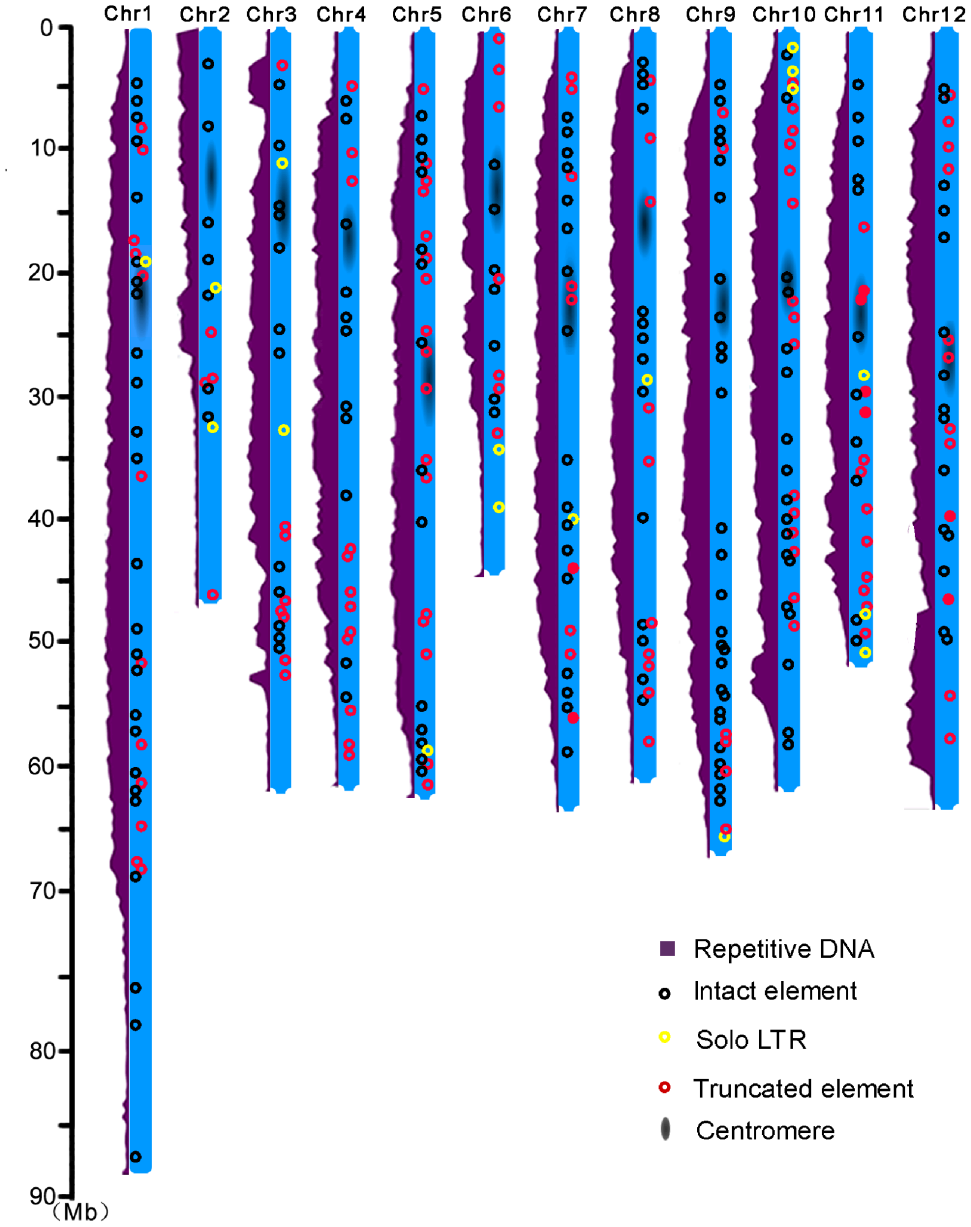
The distribution of *TARE1* elements along 12 tomato chromosomes. Each chromosome is represented by a vertical blue box. The insertions and the total repetitive DNA are marked by circles and purple regions, respectively. The potential centromeric regions are indicated by a black blur in the middle [16].

Although most LTR-RT families were found to insert into highly heterochromatic regions [22], [23], [24], there are some exceptions. For instance, *SMARTs*, the presumed smallest LTR-RTs found to date, were distributed throughout the genomes and were often located within or near genes [25]. Since the tomato pericentromeric heterochromatin comprises ∼80% of the genomic DNA [16], [17], we were interested to see if the distribution patterns of the *TARE1* elements had any difference between the two contrasting genomic environments, heterochromatic regions and euchromatin regions. Thus, we calculated the density of *TARE1* elements in the euchromatin, heterochromatin, and predicted centromeres. As expected, most of the *TARE1* elements were found to be located in heterochromatin, and exhibited apparent enrichment between the euchromatin and the heterochromatin (*p*<4.0×10^−3^, Figure 2). The difference between the density of *TARE1* elements within the euchromatin and the predicted centromeric regions was not statistically significant (*p* = 0.47, Figure 2), indicating that the *TARE1* elements are not enriched in centromeres, and they do not belong to centromeric retrotransposons.

### Most *TARE1* Elements are Shared between Cultivated Tomato and Wild Tomato

The recent release of the draft sequence of *S. pimpinellifolium*, the closest wild relative of cultivated tomato *S. lycopersicum*, allowed for a comparative analysis of *TARE1* elements between the two genomes [26] (Figure S2). Assuming that each *TARE1* insertion site is unique, we should be able to estimate the status (presence/absence) in its wild relative *S. pimpinellifolium*. For each *TARE1* insertion in *S. lycopersicum*, two unique 100-bp sequences, each composed of 50-bp of one retrotransposon terminal sequence and 50-bp of flanking DNA, were extracted and used to search against the draft genome sequence of *S. pimpinellifolium* (see Materials and Methods). The insertion of a *TARE1* element was considered to be shared between *S. lycopersicum* and *S. pimpinellifolium* if at least one junction sequence could be found in the latter. Otherwise, the insertion was considered to be unique in the former.

Using the above methodology, we analyzed 302 *TARE1* elements, including 180 intact elements with TSDs, 10 solo LTRs with TSDs, and 112 truncated elements with at least one complete LTR (Table S1 and Figure S3). Other *TARE1* elements without TSDs were not analyzed, because these elements were believed to have undergone one or more complex recombination events [14], [15]. The data showed that 252 (161 intact elements, 7 solo LTRs, and 84 truncated elements) *TARE1* insertions were shared between *S. lycopersicum* and *S. pimpinellifolium*, indicating that the majority of the *TARE1* elements (∼83%) were inserted before the split of *S. lycopersicum* and *S. pimpinellifolium* from a common ancestor (Figure S3). However, this value may still be underestimated since the *S. pimpinellifolium* genome was not well assembled and a large proportion of repetitive DNA may not be anchored to the genome [16]. Thus we can not role out the possibility that a small proportion of unshared *TARE1* insertions may be actually caused by the missing and/or wrong assembly of the *S. pimpinellifolium* genomic DNA.

### Variable Spectrum of Activity for Amplification of *TARE1* Elements Over Evolutionary Time

**Figure 3:**
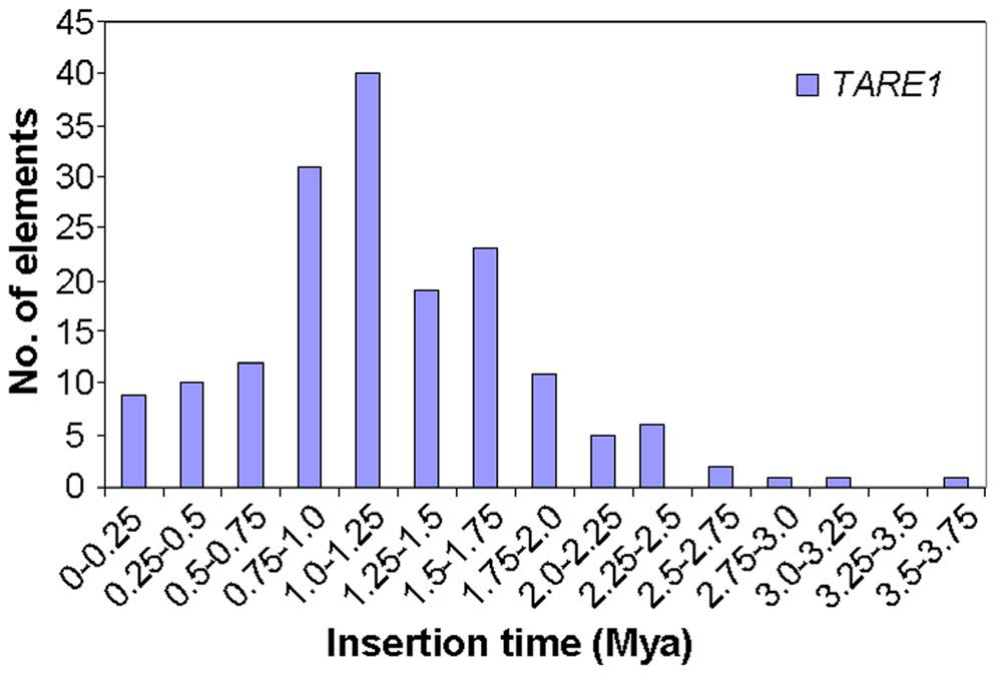
Timing and activities of *TARE1* amplification in tomato.

Since the two LTR sequences of an LTR-RT element are identical at the time of insertion, and then diverge and accumulate mutations independently, the sequence divergence of two LTRs of a retrotransposon can be converted to the insertion time of the element [7]. As anticipated, most LTR-RTs were amplified in the last 1 Mys, and LTR-RTs with age >5 Mys were rare [8], [15], as intact LTR-RTs have been rapidly changed to solo LTR, truncated elements, or completely removed from the genome over evolutionary time [2], [8]. To determine the spectrum of activity for *TARE1*, we employed the LTR-RT evolutionary rate 1.3×10^−8^ per site per year, which has been used for monocot rice [15], eudicot soybean [15], and wild tomato [26], and dated 171 intact elements in cultivated tomato. The data showed that most *TARE1* elements (66%) were inserted in the genome during 0.75–1.75 Mys, and only a small part of the *TARE1* elements could be dated <0.75 Mys (18%) or >1.75 Mys (16%) (Figure 3). A total of 40 *TARE1* elements (23%) had the highest activity within the time frame 1–1.25 Mys (Figure 3). These results suggest that *TARE1* has variable activity for proliferation within the last 4 Mys, and it has a relatively short burst of activity within the last 0.75–1.75 Mys (Figure 3). Furthermore, we only found one *TARE1* element with age 0 Mys (Table S1). However, there is a 14-bp indel between the two LTRs, indicating that this element was not inserted into the genome currently (Table S1). The evidence that none of the tomato EST sequences match *TARE1* further indicates that this family may not be active now.

### Divergence time between *S. lycopersicum* and *S. pimpinellifolium*

Assuming that the genomic sequences of cultivated tomato (*S. lycopersicum*) and wild tomato (*S. pimpinellifolium*) are identical when the two species were split, the divergence time between them can be roughly estimated on the base of the nucleotide divergence and an appropriate neutral evolutionary rate. By using a rate of 6.03×10^−9^ synonymous substitutions per site per year [27], Nesbitt and Tanksley (2002) suggested that the genus *Lycopersicon* began its initial radiation >7 Mya and that *S. lycopersicum* and *S. pimpinellifolium* diverged from a common ancestor ∼1.3–1.4 Mya depending on different cultivars investigated [28]. A recent study also indicated that the divergence at *SUN* locus between the two species occurred ∼1.6 Mya [19]. However, these estimates might have been overestimated since both genomes accumulated mutations independently after split, and ‘2T’ time instead of ‘T’ time has elapsed since the divergence of two species from a common ancestor (‘T’ indicates the divergence time from a common ancestor, see Materials and Methods).

In an attempt to further understand the divergence time between *S. lycopersicum* and *S. pimpinellifolium*, we first aligned the orthologous LTR sequences for each shared *TARE1* insertion. Using the same LTR-RT substitution rate (1.3×10^−8^ mutations per site per year), we calculated the divergence time of 153 shared orthologous LTRs. The data showed that 131 *TARE1* loci (86%) were dated <0.5 Mys (Table S1). On an average, the divergence time was estimated to be ∼0.28 Mya. For comparison, we also reanalyzed 120 intact *Rider* elements, 81 (67%) of which were found to be shared between *S. lycopersicum* and *S. pimpinellifolium* (Table S2). We found that a total of 71 (89%) shared *Rider* loci could be dated <1 Mys, and the average divergence time for 81 *Rider* loci was ∼0.46 Mya (Table S2). These results are also consistent with the analysis from the tomato whole genome level [16]. Assuming that the average substitution rate for the tomato genome sequence ranges from 6.03×10^−9^ mutations per site per year (for nuclear genes, [27]) to 1.3×10^−8^ mutations per site per year (for LTR-RTs, [15]), the average 0.6% nucleotide divergence between *S. lycopersicum* and *S. pimpinellifolium* [16] was converted to 0.23–0.5 Mya. These observations suggested that the split between *S. lycopersicum* and *S. pimpinellifolium* occurred quite recently (most likely <0.5 Mys).

Although the domestication time of tomato is not clear yet, the data from other species indicated that most cultivated crop species, including rice [29], [30], maize [31], and soybean [32], have only a few thousand years history. Thus it is not difficult to conclude that the divergence time between *S. lycopersicum* and *S. pimpinellifolium* predated the domestication of tomato.

### Species-specific Amplification of *TARE1* in Tomato, but not in Potato

**Figure 4:**
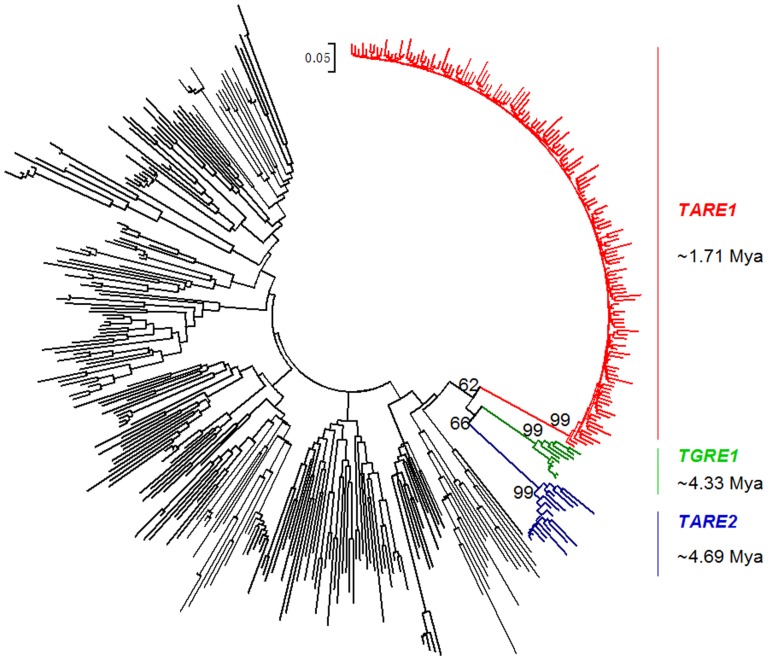
Phylogenetic tree based on the nucleotide sequences from a conserved *copia*-like RT domain. The RT sequences from tomato, rice, soybean and *Arabidopsis* were aligned using the MUSCLE program, and then the tree was reconstructed using the MEGA 5 program (see Materials and Methods). For a better visualization, only the elements from tomato *TARE1*, *TGRE1*, and *TARE2* families, and exemplars from other species are shown.

As we mentioned earlier, one feature of *TARE1* is the presence of dinucleotides ‘TA’ at the beginning of both LTRs. In an attempt to track the origin of *TARE1*, we performed a phylogenetic analysis using a conserved RT domain of intact LTR-RTs in tomato, as well as *Copia*-like LTR-RTs in *Arabidopsis*, rice and soybean, identified previously [8]. Interestingly we found that two other families, *TARE2* and *TGRE1* were phylogenetically close to *TARE1* (Figure 4). The three families all belong to *Ale* lineage, but formed a distinct sublineage, which was distinguishable from other families (Figure 4). Similar to *TARE1*, the two LTRs of *TARE2* also started as ‘TA’. Nevertheless, *TGRE1* does not share such a characteristic (Figure S4). In addition, detailed annotation for the internal region revealed the complex structures of *TARE1*. Although the majority of *TARE1* elements contain a full set of genes necessary for transposition, some lack *gag*, *int*, and/or *rt* genes, indicating that these are incomplete copies of *TARE1* (Figure S4).

Assuming that all elements in a LTR-RT family are generated from a common ancestor, the sequence divergence level of LTRs with the ancestor LTR can reflect the time elapsed since the last common ancestor (the age of family) [23]. In practice, the ancestor copy may not be recognizable, or could have been removed from the genome. Thus the consensus sequence of all elements usually represents the status of the common ancestor [23]. Using the same LTR-RT evolutionary rate 1.3×10^−8^ per site per year [15], we estimated the age of all the three LTR-RT families. Our data showed that *TARE1* family was the youngest group, and was dated at 1.71 Mya. *TGRE1* and *TARE2* families were relatively older, at 4.33 Mya and 4.69 Mya, respectively (Figure 4). The fact that none of these three families can be found in potato, suggests that they might have been specifically amplified in the tomato genome after speciation.

### Conservation, Divergence, and Differential Amplification of *TARE1*, *TARE2*, and *TGRE1*

**Figure 5:**
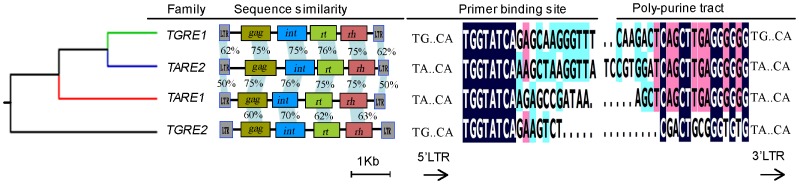
Phylogenetical relationship and sequence similarity between *TGRE1*, *TARE2*, and *TARE1*. The phylogenetically closest family *TGRE2* was set as an outgroup. The physical positions of each representative element are located at chromosome 1 from 27235875 to 27240535 for *TARE1*, chromosome 7 from 61990209 to 61994987 for *TARE2*, chromosome 5 from 23621836 to 23626419 for *TGRE1*, and chromosome 11 from 7616310 to 7621220 for *TGRE2*, respectively.

Phylogenetic tree usually reflects the relationship between different families. Using the phylogenetically closest tomato family *TGRE2* as outgroup, the evolutionary relationship between *TARE1*, *TARE2*, and *TGRE1* has been established (Figure 4). Following a unified classification for eukaryote transposable elements, *TARE1*, *TARE2*, and *TGRE1* were grouped into three distinct families. As illustrated in Figure 4, *TARE2* and *TGRE1* are closely related, not only because they have similar element size, but also because they both share substantial sequence similarity in LTR regions, internal polyprotein, primer binding site, and polypurine tract (Figure 5). In contrast, *TARE1* has a smaller element size, and shares lower sequence similarity with *TARE2* than *TGRE1* does. However, LTR sequences generally diverge faster than the coding polyprotein, since the former usually exhibits lower sequence similarity (Figure 5).

Although the three families are closely related, the timing, scale, and activity of amplification are quite different (Figure 4). For instance, *TGRE1* has the lowest copies; however, 4 out of 10 intact elements have two identical LTRs, indicating that it may still be active now (Figure 4, Table S3). In contrast, the youngest *TARE2* was generated within the last 0.66 Mys, and ∼50% of *TARE2* elements had activity during the last 2–3.5 Mys (Table S3). It is particularly interesting that only *TARE1* has dramatically proliferated in tomato within the last 2 Mys. However, the molecular mechanism for the burst of *TARE1* remains unclear, and needs further clarification.

## Discussion

### *TARE1*, a Mutated LTR Retrotransposon in the Tomato Genome

The annotation for LTR-RTs mainly relies on structure-based programs, such as LTR_STRUC [21] or related programs [33], [34], particularly when the genome sequence is new and the reference TE database is not available. However, LTR_STRUC cannot detect more than one third of the LTR-RTs in a genome [3]. In this study, only 18 out of 354 *TARE1* elements (∼5%) were identified by the LTR_STRUC. In addition, the elements identified without “TG..CA” in the termini were often regarded as wrong annotations and were not analyzed further [35]. Thus, it is not surprising to see that most LTR-RT families described in plants share highly conserved structures, including dinucleotide “TG” at the beginning of both LTRs. One exception is *Tos17*, a well investigated LTR-RT family in rice, which contains “TG..GA” at two LTRs [36]. However, *Tos17* has only two copies in the sequenced *japonica* rice genome, and while it contains 1–5 copies in other rice cultivars under normal growth condition [37]. Thus, the impact of *Tos17* on the structure and evolution of the rice genome is limited. In this study, >300 *TARE1* elements share the same structure as “TA..CA”, indicating that LTR-RTs with atypical structure can be substantially amplified in the host genome. The new data will provide a valuable addition to tens of thousands of typical LTR-RTs in the tomato genome, and will also provide hints for the complete annotation of other genomes.

### Using *TE*-junction Markers as an Alternative Approach to Estimate the Divergence time between Tomato and its Wild Relative

TEs are abundant and highly variable within species, subspecies, and cultivars. For instance, transposon insertion polymorphisms contribute ∼14% of the genomic DNA sequence differences in *indica* and *japonica* [38]. Recently, using a semi-automated bioinformatics pipeline, Tian and his colleagues identified 34154 non-redundant TE insertions in 31 resequenced soybean genomes [10], [39]. However, only 5731 TE insertions (17%) were detected in the 14 cultivated accessions. On an average, 2100 TE insertion differences occur per accession [10].

TEs are not only a valuable resource for structural variations in plant genomes, but can also be used as molecular markers to track the evolutionary history. They are also potentially useful for estimation of the divergence time between cultivated and wild crop species. Nevertheless, compared with using synonymous sites (Ks) in coding genes as markers for calculation [16], [28], [37], the use of TEs poses some difficulties in estimating the divergence time between cultivated and wild species: (1) TEs are highly repetitive in genomes, and the accurate assembly of TEs is not easy; (2) the genomic sequence of the wild relative is often unavailable; (3) TEs evolve very fast in a genome, and many of them are truncated or unrecognizable. Thus, TE-based estimation for dating the split time of two species should meet at least three qualifications: (1) the availability of a high-quality assembled genome sequence for TE identification; (2) enough information on genomic DNA of its wild species for TE-junction comparison; (3) the presence of two closely related species (ideally <1 Mys), which could be used for shared and unshared TE analysis. In this study, we performed the first ever genome-wide searches for a single LTR-RT family in tomato, and identified 354 *TARE1* elements, ∼83% of which were shared between cultivated and wild tomato. Using the shared *TARE1* elements, we estimated that the divergence time between the two species was ∼0.28 Mya. This value was about five times younger than previously reported [19], [28]; however it was close to the split time (0.27 Mys) suggested between cultivated and wild soybean [40]. We also compared our data with Ks-based estimation for the split time between cultivated and wild tomato. In 31859 orthologous gene comparisons, the average synonymous substitution (Ks) for *S. lycopersicum* and *S. pimpinellifolium* is 0.0052 (range from 0 to 0.1864) [16]. We applied an evolutionary rate of 6.03×10^−9^ substitution per site per year for *Adh* gene [27] to estimate the split time based on Ks analysis between two species at 0.43 Mya, an estimate which is very close to the *Rider TE*-based estimation in this study (0.46 Mya). Although the substitution rates in different species may be slightly different [2], the nucleotide divergence estimated from the whole genome level suggests a relatively younger split time between *S. lycopersicum* and *S. pimpinellifolium* during the last 0.23∼0.5 Mya [16]. Therefore, the divergence time between *S. lycopersicum* and *S. pimpinellifolium* had occurred approximately <0.5 Mys, which is much younger than the previously estimated time [19], [28].

Since the domestication for most major crops occurred only within about ten thousand years, the domestication of tomato might be more complicated than expected. The ancestor of the cultivated tomato probably occurred and evolved for a long time, particularly at an early stage after speciation, similar to the domestication of soybean [40].

### The Molecular Mechanism Responsible for the Unique Structure and Proliferation of *TARE1*

It has been well documented that TEs are ubiquitous in plant kingdoms; however, the majority of them turn out to be silent under normal growth conditions, and are unable to amplify further in their host genome. Only a small proportion of TEs have transcriptional and/or translational activities. This is partially because of the occurrence of substantial mutations, frameshifts, and stop codons in the coding regions. Although the two LTRs of an element do not contain any genes related with the transpositional process, they do include three regions comprising *cis*-elements for the transcription start and termination, and for the integration of the element [19]. The transcription of a LTR-RT element usually initiates at the 5′ start of R in the 5′LTR and terminates in the 3′ end of R in the 3′ LTR [1]. Thus, the genetic changes in LTR regions may affect their transcriptional activities.

Several lines of evidence indicate that *TARE1* is a mutated LTR-RT family, given the fact that the two LTRs of *TARE1* terminate with ‘TA’ rather than ‘TG’, and the amplification of *TARE1* seems to be species-specific in tomato. Furthermore, the *TARE1* sublineage appears to evolve from other LTR-RT families with dinucleotides “TG”. The evidence that both LTRs of *TARE1* contain ‘TA” and that *TARE1* contains >300 copies, suggests that neither transcriptional nor translational process was interrupted after mutation. This finding is quite similar to our previous report on *SNRE^S^* subfamily in soybean, which carries a foreign solo LTR in the internal part, but is dramatically amplified in the soybean genome [41]. However, we are not certain whether this single mutation has any correlation with the amplification of *TARE1*. Since *TARE1* is an autonomous element, and the majority of its copies contain complete structure as other elements like *Rider*, thus, its amplification does not need enzymes encoded by other elements, as suggested for non-autonomous *Dasheng* in rice [23] and *SNRE^S^* in soybean [41].

**Figure 6:**
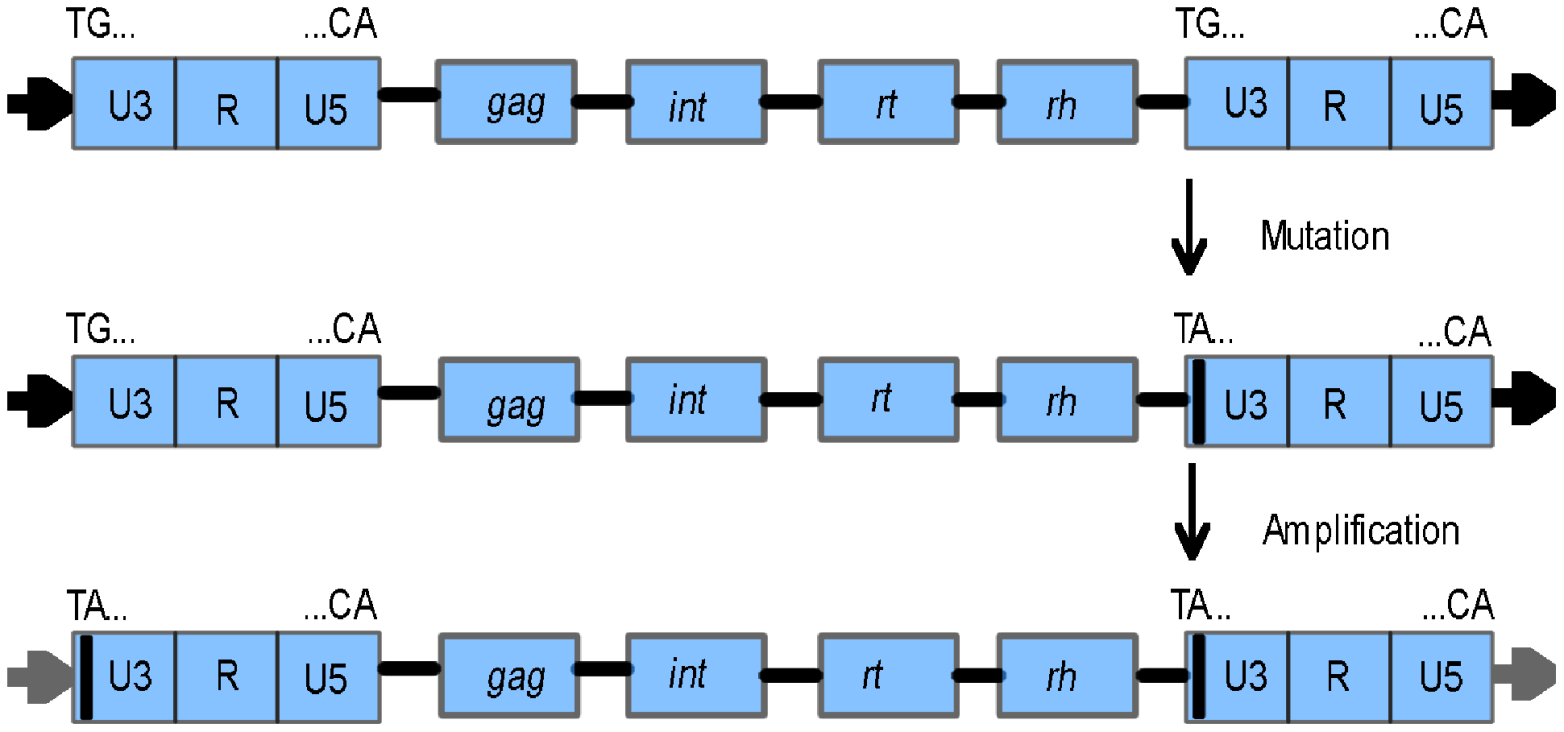
A model for the evolution and amplification of *TARE1*. The horizontal arrows flanking the elements indicate the target site duplications (TSDs). The black line within the U3 of LTR indicates the mutation from ‘G’ to ‘A’.

There are several possible explanations regarding the origin of *TARE1*, of which one might be the “genomic DNA mutation hypothesis”. Of course, a single mutation from ‘G’ to ‘A’ at both LTRs would yield the structure of *TARE1*, as illustrated in Figure 1. However, the chance of generating the same mutation at the same site seems pretty low. The possibility that a few hundred copies share the structure of *TARE1* also appears unlikely. The second hypothesis is the 5′ LTR mutation from ‘G’ to ‘A’ followed by the transposition of *TARE1*. Although there is no direct experimental evidence about the regeneration of a LTR-RT in plants, the process was believed to be quite similar to that of retroviruses [2]. Nevertheless, based on the knowledge of retrovirus transposition process, the following copies would be recovered to the original ones, making this hypothesis impractical. Alternatively, it could be caused by the 3′ LTR mutation from ‘G’ to ‘A’ followed by the transposition of *TARE1*. According to this, the following copies generated from the mutated *TARE1*, would all carry this mutation at both LTRs, as was observed in this study (Figure 6).

Theoretically, the ancestral copies of *TARE1* elements without mutation can continue to amplify following the mutation in one copy. If this deduction were true, we would expect many or at least a few copies of *TARE1* with ‘TG..CA” at both LTRs. However, in the entire tomato genome, no element shared such a structure, even for partially deleted truncated copies. One possibility may be that one ancestor copy without mutation had evolved into another family, like the *TGRE1* (Figure 4). The evidence that the two LTRs of *TARE2* also terminate with “TA..CA”, indicates that at least two copies with this G->A mutation were regenerated via RNA process.

Further investigation of atypical LTR-RTs in other sequenced plant species might provide interesting insights into their structural evolution. The ongoing comparative analysis from multiple species will facilitate our understanding of the frequency of occurrence of these mutated LTR-RTs, and the way they affect the gene and genome evolution in the context of their evolutionary history.

## Materials and Methods

### Genome Sequence Data and Identification of LTR-RTs

The assembled tomato (*S. lycopersicum*) genome sequence (V2.40), the scaffolds of wild tomato (*S. pimpinellifolium*) genome sequence and the assembled potato genome sequence used in this study are publicly available and downloadable at the *SGN* website (http://solgenomics.net/). The LTR-RTs were identified by a combination of structural analysis and sequence homologous comparisons [4], [15]. Initially the *LTR_STRUC* program was employed to search the relatively young intact elements [21], and the missed intact elements; the solo LTRs and truncated elements were identified by the *cross_match* program with the default parameters [4], [15].

### Strategy to Define Shared and Unshared LTR-RTs between Species

To define shared and unshared LTR-RTs, a modified strategy from a previous approach was employed [42]. Briefly, the process included the following steps: (1) extracting one or two 100-bp LTR-RT junction sequences for each element in *S. lycopersicum*, including 50-bp flanking sequences and 50-bp LTR-RT terminal; (2) using the 100-bp sequences as queries, to do a *cross_match* with the default parameters, and to search against the scaffold sequences of *S. pimpinellifolium*; (3) a shared element was defined when at least one site of 100-bp sequence could be found in *S. pimpinellifolium*. Otherwise, the element was considered to be unshared between two species.

### Estimation of Insertion Time

Intact elements with two complete LTR sequences were aged by comparing the divergence of their 5′ and 3′ LTRs. For each element, two LTRs were aligned by using the program MUSCLE with default parameters [43]. The insertion time (T) for a given intact LTR-RT element was calculated using the formula: T = K/2r. Kimura-2 parameter distances (K) between 5′ and 3′ LTRs were calibrated by the Jukes-Cantor method [44]. The *r* represents an average substitution rate of LTRs, which is 1.3×10^−8^ substitution per site per year [45].

The ages or insertion times (T) of *TARE1*, *TARE2* and *TGRE1* (phylogenetic groups) since the divergence from each group’s common ancestor, were estimated using the formula: T = K/r [23]. The average Kimura 2-parameter distance (K) was calculated by the alignment of each intact element in a group with the consensus sequence of that group [46], [47]. The cutoff of consensus sequences was 50% which was determined from the EMBL consensus sequence server (http://coot.embl.de/Alignment//consensus.html). The average mutation rate of LTRs is 1.3×10^−8^ substitution per site per year [45].

### Phylogenetic Analysis

A typical *Copia*-like conserved RT domain sequence was set as a tblastn query, to search against the *TARE1*, *TARE2* and *TGRE1* intact element database (E-value <10^−9^). The cDNA sequences of RT domains were extracted to align together with other 200 *Copia*-like RT domain DNA sequences from soybean, rice and *Arabidopsis* using MUSCLE program with default options. The phylogeny of this alignment was reconstructed using the bootstrap neighbor-joining method [48] with Kimura 2-parameter method implemented in the MEGA 5 program [49].

## Supporting Information

Figure S1 **Alignment of LTR sequences and annotation for the three parts of a** ***TARE1*** **LTR.** The predicted U3, R and U5 regions are indicated between the arrows. The 6-bp nucleotides within the R region were presumed to be related with polyadenylation and the 4-bp nucleotides within the U5 region were considered to be important in termination of RNA synthesis. The 12 intact elements were selected randomly and the physical positions for each element (from the top to the bottom) are Chr7_13821723_13826394, Chr11_30046017_30050224, Chr12_6018215_6022911, Chr3_14288754_14293444, Chr9_8098937_8103667, Chr6_20486319_20490978, Chr1_5417021_5421735, Chr5_9416240_9421026, Chr8_7111971_7116630, Chr10_20942005_20946722, Chr2_29429777_29434486, and Chr4_30724922_30729638.
(TIF)

Figure S2 **Phylogenetic relationships and divergence time between 4** ***Solanum*** **species,** ***Petunia inflate*** **, and** ***Arabidopsis thaliana*** **.** The tree was modified based on a previous study [26]. The divergence time between *S. lycopersicum* and *S. pimpinellifolium* was suggested in this study.
(TIF)

Figure S3 **Shared and unshared** ***TARE1*** **elements between** ***S. lycopersicum*** **and** ***S. pimpinellifolium*** **.** The intact elements with TSDs (A), solo LTR with TSDs (B), and truncated elements with at least one complete terminal (C) were investigated (see Materials and Methods). As the *S. pimpinellifolium* genome has not been well assembled yet, the unshared *TARE1* elements in its genome were not analyzed, and are indicated by the question mark here.
(TIF)

Figure S4 **Structural annotation for** ***TARE1*** **,** ***TARE2*** **and** ***TGRE1*** **.** LTR, long terminal repeat; PBS, primer binding sites; PPT, polypurine tracts; *gag*, group-specific antigen gene; *int*, integrase; *rt*, reverse transcriptase; *rh*, RNAase-H.
(TIF)

Table S1 **Summary of** ***TARE1*** **elements identified in the tomato genome.**
(XLS)

Table S2 ***Rider*** **intact elements identified in this study.**
(XLS)

Table S3 ***TARE2*** **and** ***TGRE1*** **elements identified in this study.**
(XLS)
