# Supplementary figures and images for: TARE1, a Mutated Copia-Like LTR Retrotransposon Followed by Recent Massive Amplification in Tomato
Source: PLoS One. 2013 Jul 4;8(7):e68587. doi: 10.1371/journal.pone.0068587 (PMC3701649; doi:10.1371/journal.pone.0068587)

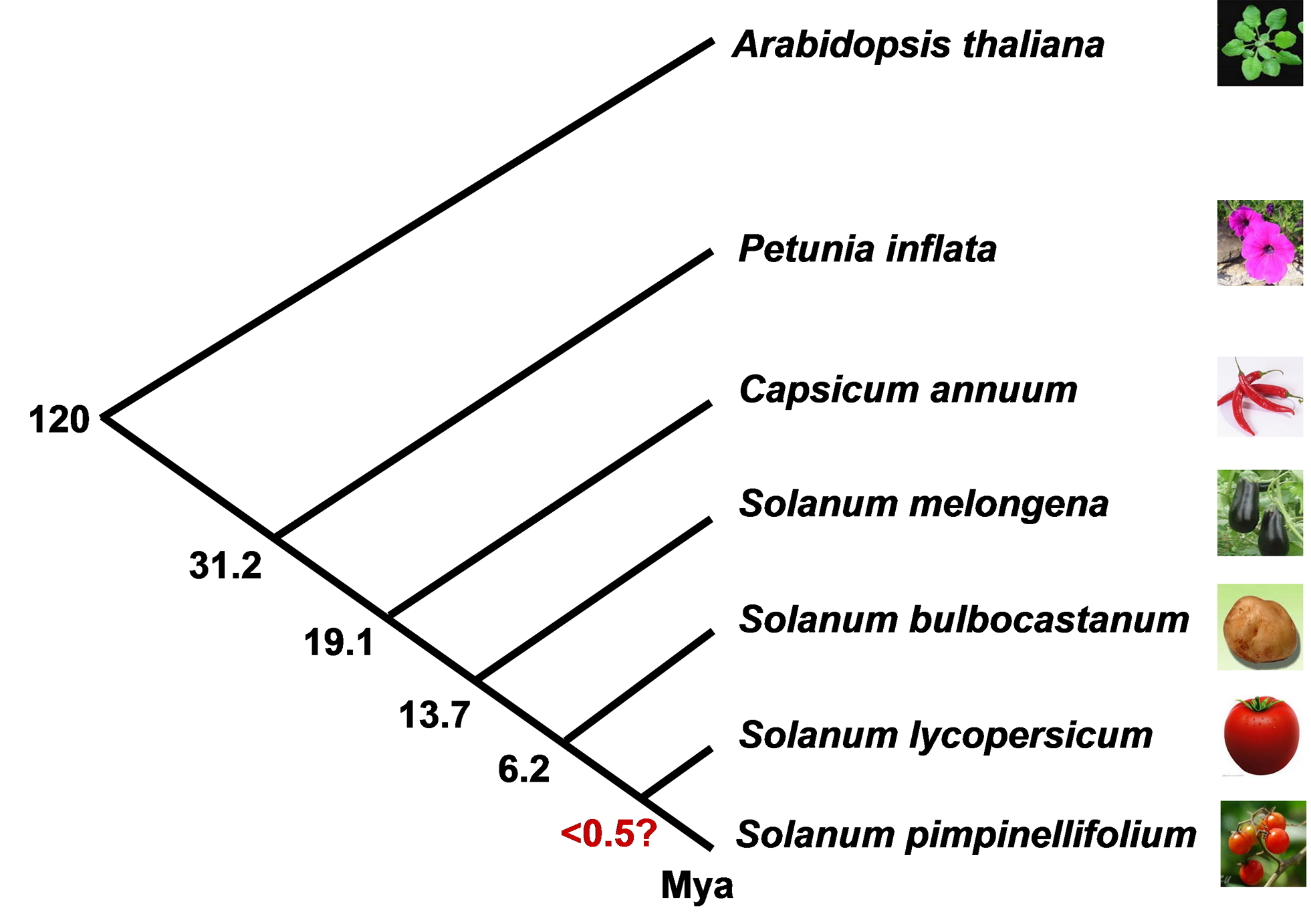

Supplement: Figure S2 — Phylogenetic relationships and divergence time between 4 Solanum species, Petunia inflate , and Arabidopsis thaliana . The tree was modified based on a previous study [26]. The divergence time between S. lycopersicum and S. pimpinellifolium was suggested in this study. (TIF) [file pone.0068587.s002.tif]
